# Meta-analysis for the associations of serum C-reactive protein with delirium risk

**DOI:** 10.3389/fneur.2026.1728476

**Published:** 2026-01-29

**Authors:** Qi-xian Liang, Xiao-li Tan, Zhi-wei Huang, Shuai Chen, Ye-zhao Li, Zhao-yin Fu

**Affiliations:** Department of Intensive Care Unit, Tenth Affiliated Hospital of Guangxi Medical University, Qinzhou, China

**Keywords:** association, C-reactive protein, delirium, meta-analysis, serum

## Abstract

**Background:**

This meta-analysis aimed to evaluate the association between serum C-reactive protein (CRP) levels and delirium risk, encompassing postoperative delirium (POD) and delirium secondary to other medical conditions.

**Methods:**

A systematic search was conducted across PubMed, Web of Science, and the Chinese National Knowledge Infrastructure database. The odds ratio (OR) with their 95% confidence intervals (CI) from each study were extracted and used to estimate the effects. Meta-regression analysis was utilized to identify potential sources of heterogeneity. Subgroup analyses were applied to explore the association under different disease types (POD vs. non-POD), and age groups (>70 years or ≤70 years) among delirium patients.

**Results:**

A total of 9,002 patients from 32 included studies were analyzed in this meta-analysis. Of these, 21 studies comprising 5,006 patients examined the association of CRP with delirium using continuous data, while 11 studies involving 3,996 patients employed categorical data by dividing patients into high and low groups based on the CRP value. The pooled data from continuous data showed that CRP levels were significantly associated with delirium (OR: 1.10, 95% CI: 1.01–1.20, *p* = 0.030); and the pooled data from categorical data revealed that high CRP levels increased the risk of delirium (OR: 2.66, 95% CI: 2.00–3.53, *p* < 0.001). Significant heterogeneity was found across the studies; however, meta-regression analysis did not demonstrate that variables such as age, study location, study design, disease type, and diagnostic criteria were primary sources of heterogeneity. Subgroup analysis indicated that CRP levels were associated with an increased risk of delirium regardless of disease type and age group.

**Conclusion:**

Elevated serum CRP levels are significantly but modestly associated with delirium risk in diverse clinical populations. Given the observational nature of the included studies and high heterogeneity, these findings support CRP as a correlate, rather than a causal mediator, of delirium-related inflammation.

## Introduction

C-reactive protein (CRP), a classical acute-phase reactant synthesized by hepatocytes in response to interleukin-6 (IL-6), has long been recognized as a key biomarker of systemic inflammation (1). Elevated CRP levels are associated with infectious, traumatic, and inflammatory conditions, reflecting the activation of innate immune pathways (2). In recent years, its relevance has expanded into neuropsychiatric research, particularly in disorders involving neuroinflammation, such as delirium (3). Delirium is characterized by acute and fluctuating disturbances in attention, awareness, and cognition, and represents a significant complication in hospitalized patients (4, 5). Among surgical populations, postoperative delirium (POD) is especially prevalent. The condition’s multifactorial etiology includes neurotransmitter imbalances, oxidative stress, and systemic inflammation, with CRP emerging as a potential mediator due to its ability to cross the blood–brain barrier and exacerbate neuroinflammatory pathways (6). Despite increasing interest, the relationship between serum CRP levels and delirium risk remains inconsistently defined, highlighting the need for a systematic synthesis of existing evidence.

Over the past decade, observational studies have reported conflicting findings regarding this association. In surgical settings, elevated preoperative or early postoperative CRP levels have been linked to an increased incidence of POD, particularly in cardiac and orthopedic surgeries. For instance, a prospective cohort study of elderly patients with hip fractures found that high preoperative CRP levels were associated with a greater risk of developing POD (7). In contrast, studies involving non-surgical populations, such as critically ill patients with sepsis or respiratory failure, have reported no significant association after adjusting for confounders like age and comorbidities (8). These discrepancies may stem from variations in the timing of CRP measurements and population-specific inflammatory profiles. Consequently, clarifying the association between CRP levels and delirium risk is crucial for guiding timely and effective interventions in at-risk patients.

Despite extensive research on CRP, its role in delirium remains poorly understood, largely due to fragmented evidence and methodological inconsistencies across studies. Meta-analysis offers a quantitative approach to synthesize data from individual studies, providing more consistent and reliable insights than isolated investigations. This meta-analysis aims to evaluate the association between serum C-reactive protein and delirium risk in clinical populations. By integrating data from diverse populations and etiologies, this work seeks to bridge the divide between biomarker research and clinical applications, ultimately reducing the burden of delirium among vulnerable populations.

## Methods

### Search strategy and study selection

We conducted a systematic literature search in PubMed, EMBASE, Ovid MEDLINE, the Chinese National Knowledge Infrastructure (CNKI), and the Chinese Biomedical Literature Database (CBM) from January 1, 2000 to March 1, 2025. The search was restricted to human studies published in English or Chinese. No other language restrictions were applied beyond these two due to resource and translation constraints, which we acknowledge as a potential limitation. In PubMed, the search strategy combined Medical Subject Headings (MeSH) terms and free-text keywords using Boolean operators as follows: (“Delirium” [MeSH Terms] OR “Acute Confusional State” [Title/Abstract] OR “Postoperative Delirium” [Title/Abstract]) AND (“C-Reactive Protein” [MeSH Terms] OR “CRP” [Title/Abstract] OR “Inflammatory Biomarkers” [Title/Abstract]). Similar strategies were adapted for EMBASE, Ovid MEDLINE, CNKI, and CBM using database-specific controlled vocabularies. Full search syntax for all databases is provided in Supplementary Table S1. To minimize omission, we also used the PubMed “Related Articles” function and manually screened reference lists of all included studies and relevant reviews for additional eligible publications. This meta-analysis adhered to the guidelines outlined in the Preferred Reporting Items for Systematic Reviews and Meta-Analyses (PRISMA) statement, with titles/abstracts independently screened by two reviewers, followed by full-text assessment for eligibility.

### Inclusion and exclusion criteria

This meta-analysis followed the PICOS principles recommended by the PRISMA guidelines (9), with clearly defined inclusion and exclusion criteria. Population (P): The study included clinical patients diagnosed with delirium. Intervention (I): The patients were included irrespective of surgical status. Control (C): The patients in control group were not diagnosed with delirium. Outcome (O): The main outcome was serum CRP levels and delirium risk. Study design (S): Observational studies were included. Exclusion criteria: Studies were excluded if they met any of the following conditions: (1) unpublished, reviews, guidelines, letters, case reports, conference abstracts, lacked accessible full texts; (2) duplicate publications; (3) studies with incomplete raw data, studies that did not assess any outcome metrics, or studies from which outcome data could not be extracted; (4) when overlapping patient cohorts were identified, only the study with the largest sample size was included.

### Literature screening and data extraction

Two reviewers independently screened titles and abstracts, followed by a detailed independent assessment of full-text articles. Data extraction focused on obtaining ORs and 95% CIs from each study. Clinical data were extracted using the Cochrane Data Extraction Form, capturing details such as the first author, year of publication, country, sample size, patient age, delirium type, study design, and diagnostic criteria. Authors of eligible studies were contacted when necessary to obtain additional information. Disagreements during this process were resolved through consultation with a third reviewer.

### Quality assessment for the studies

The Newcastle-Ottawa Scale (NOS) was employed to assess the methodological quality of all included studies. NOS evaluates cohort selection (0–4 stars), comparability (0–2 stars), and outcome assessment (0–3 stars), making it suitable for observational studies. This approach ensures consistency with previous meta-analyses. Any discrepancies in quality assessments were resolved through discussions involving corresponding authors.

### Data synthesis and meta-analysis

For categorical variables, odds ratios (OR) with 95% confidence intervals (CI) were calculated to determine effect sizes. Heterogeneity among studies was quantified using the I^2^ statistic. A random-effects model was applied when high heterogeneity (*I*^2^ > 50%) was detected; otherwise, a fixed-effects model was used for *I*^2^ < 50%. Sensitivity analyses were conducted to identify potential sources of heterogeneity. Publication bias was assessed via Egger’s test for funnel plot asymmetry. All statistical analyses were performed using R software (version 4.2.1) with the “meta” package, setting the threshold for statistical significance at *p* < 0.05.

## Results

### Identification of included studies

**Figure 1:**
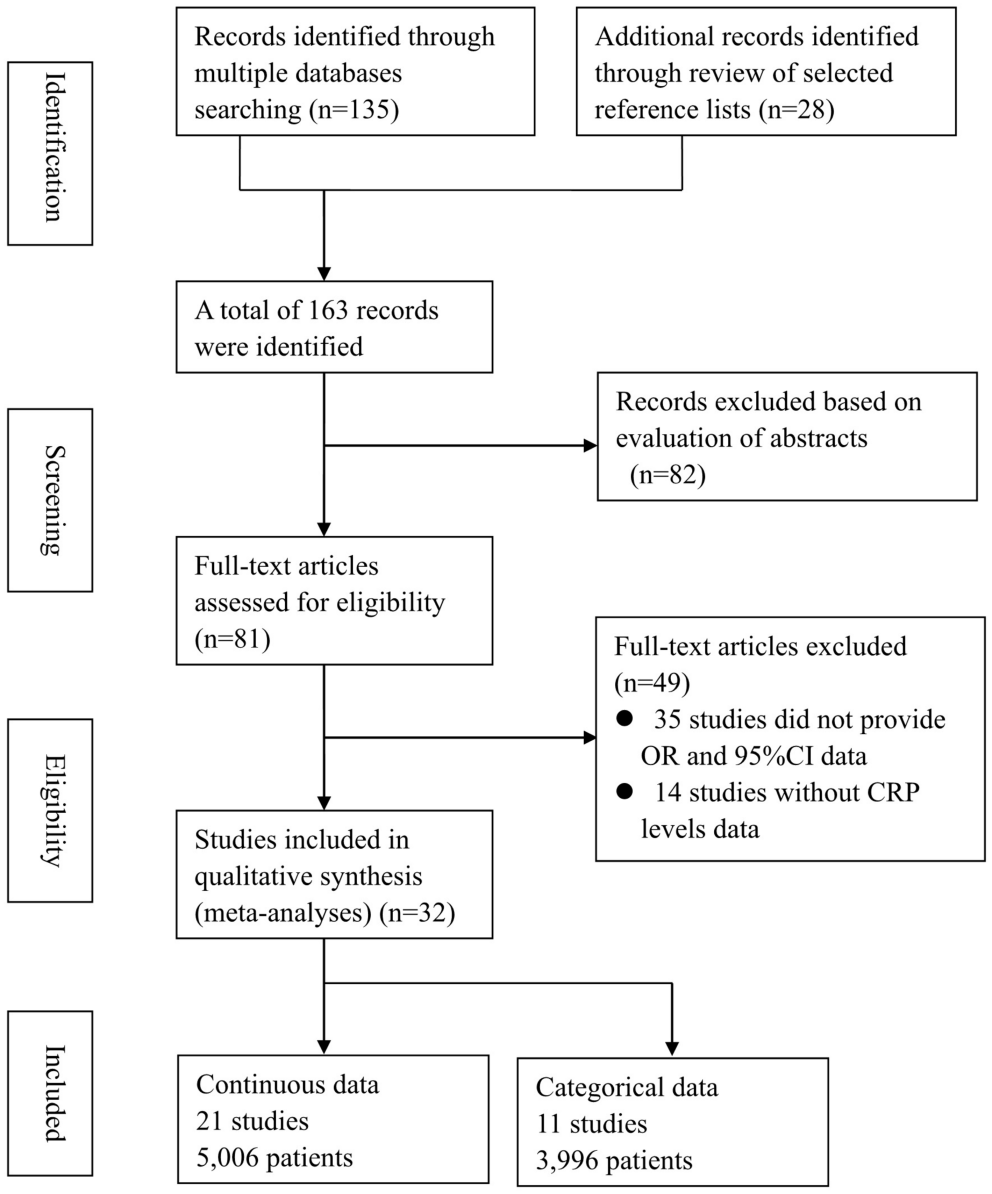
Flowchart of study selection.

A total of 163 studies were retrieved from the databases. After removing duplicates, 82 studies underwent title and abstract screening, resulting in 81 eligible studies for further review. Following full-text reviews, 22 studies were excluded due to insufficient data or other reasons. Ultimately, 32 studies reporting the association between CRP levels and delirium were selected for the final meta-analysis. A detailed overview of the study selection process is provided in the PRISMA flow diagram (Figure 1).

### Characteristics of included studies

The key characteristics of the 32 included studies are summarized in Table 1. These studies encompassed a total of 9,002 patients (10–41). Among the studies, 15 adopted a prospective design, while 17 employed a retrospective approach. Geographically, 26 studies were conducted in China, and the remaining seven were from Canada, Germany, Korea, Poland, and Spain. The patient populations varied widely in terms of underlying conditions, which were broadly categorized into surgical and non-surgical groups. Patient age ranged from 57.7 to 86 years. Delirium was diagnosed using standardized criteria, including the Confusion Assessment Method (CAM), CAM for the Intensive Care Unit (CAM-ICU), and the Diagnostic and Statistical Manual of Mental Disorders (DSM-5 or DSM-IV). Of the 32 studies, 21 studies involving 5,006 patients analyzed CRP levels as continuous variables (10, 13, 15–17, 20–27, 29, 30, 33, 35–38, 40), while 11 studies involving 3,996 patients categorized patients into high and low CRP groups based on predefined thresholds (11, 12, 14, 18, 19, 28, 31, 32, 34, 39–41). Study quality was assessed using the NOS, with scores indicating moderate to high quality across all studies, with the score range from 7 to 9 (Supplementary Table S2), indicating moderate to high methodological quality overall.

**Table 1:** Characteristics of included studies.

First author	Year	Country	OR (95%CI)	Design	Diseases	Sample size	Age	Criteria	Cut-off
Kaźmierski	2021	Poland	1.015 (0.998–1.032)	P	Cardiac Surgery	177	67 (63–71)	DSM-5	
Lian	2024	China	1.002 (1.001–1.023)	P	ARDS	205	66 (56–78)	CAM-ICU	
Liao	2024	China	1.007 (1.004–1.01)	R	Pneumonia	379	80.0 ± 7.4	DSM-5	
Lozano	2024	Spain	1 (0.98–1.03)	P	Hip fracture	60	86.0 ± 6.3	DSM-5	
Ma	2022	China	1.43 (1.01–2.04)	P	Knee/hip replacement	143	71 (67–76)	DSM-IV	
Miao	2018	China	1.22 (0.92–1.69)	P	Abdominal surgery	112	71.8 ± 6.6	DSM-IV	
Qin	2025	China	1.007 (1.003–1.011)	R	General anesthesia	644	64.02 ± 13.2	CAM	
Ren	2020	China	1.047 (1.013–1.082)	P	Cervical or Lumbar Surgery	206	57.7 ± 11.3	CAM	
Zhang	2022	China	1.02 (1.005–1.034)	P	Knee arthroplasty	268	70.29 ± 5.03	CAM	
Zhou	2022	China	1.01 (0.999–1.03)	P	Parkinson’s Disease	70	63.70 ± 4.28	CAM	
Zou	2025	China	1.001 (0.999–1.004)	P	Hip arthroplasty surgery	287	76 ± 12	CAM	
Chang	2024	China	1.01 (1.002–1.018)	R	Pneumonia	397	78.66 ± 6.97	CAM	
Jin	2024	China	1.817 (1.007–3.277)	R	General anesthesia	283	71.52 ± 4.33	CAM-ICU	
Li	2024	China	0.862 (0.795–0.935)	P	Traumatic brain injury	107	60. 23 ± 10. 79	CAM-ICU	
Liu	2024	China	1.013 (0.999–1.028)	R	Bone surgery	440	81.14 ± 7.06	CAM	
Xu	2022	China	1.169 (1.049–1.302)	R	Knee/hip replacement	156	78.58 ± 8. 21	CAM	
Ye	2018	China	1.005 (0.999–1.001)	R	Pneumonia	133	70.47 ± 12.61	CAM-ICU	
Zheng	2022	China	2.125 (1.986–2.265)	R	Sepsis	352	70.15 ± 6.24	CAM-ICU	
Forget	2021	Canada	1.68 (1.23–2.4)	R	COVID-19	127	82 (74–88)	DSM-5	
Hindiskere	2020	Korea	1.17 (1.06–1.29)	P	Surgery for bone	276	64 (16–94)	DSM-IV	
Li	2022	China	1.017 (1.007–1.027)	P	Lower limb fracture	184	76.15 ± 7.94	CAM	
Kotfis	2019	Poland	2.593 (1.736–3.873)	R	Stroke	760	75.9 ± 13.5	CAM-ICU	6.1
Knaak	2019	Germany	4.771 (1.765–12.899)	P	Neurocognitive disorder	314	73 (68–77)	DSM-IV	5
Ding	2020	China	9.504 (2.143–42.15)	P	Acute Pain	60	64.5 (52–73)	CAM	2
Xiang	2022	China	1.68 (1.02–2.81)	R	Gynecologic cancers	226	70.4 ± 2.7	DSM-5	8
Zhang	2022	China	3.695 (1.958–6.973)	R	Knee/hip replacement	200	73.23 ± 3.56	CAM	8
Zhu	2023	China	2.903 (1.673–5.037)	R	Knee/hip replacement	802	80.9 ± 8.1	CAM	14
Ding	2023	China	4.524 (2.619–7.817)	R	Hip arthroplasty surgery	483	82.1	CAM	90
Sun	2023	China	1.211 (0.714–2.053)	P	Colorectal Cancer surgery	643	69.31 ± 5.12	CAM	48
He	2023	China	1.722 (0.914–3.245)	R	Hip arthroplasty surgery	426	79. 56 ± 6. 89	CAM	0.68
Pan	2024	China	1.984 (1.078–3.65)	R	Knee replacement	112	70.1 ± 4.2	CAM	8
Yan	2024	China	2.159 (1.194–3.906)	R	Intertrochanteric fracture	293	82.24 ± 8.57	DSM-IV	20.25

P, prospective; R, retrospective.

### Association of CRP levels with delirium based on continuous data

**Figure 2:**
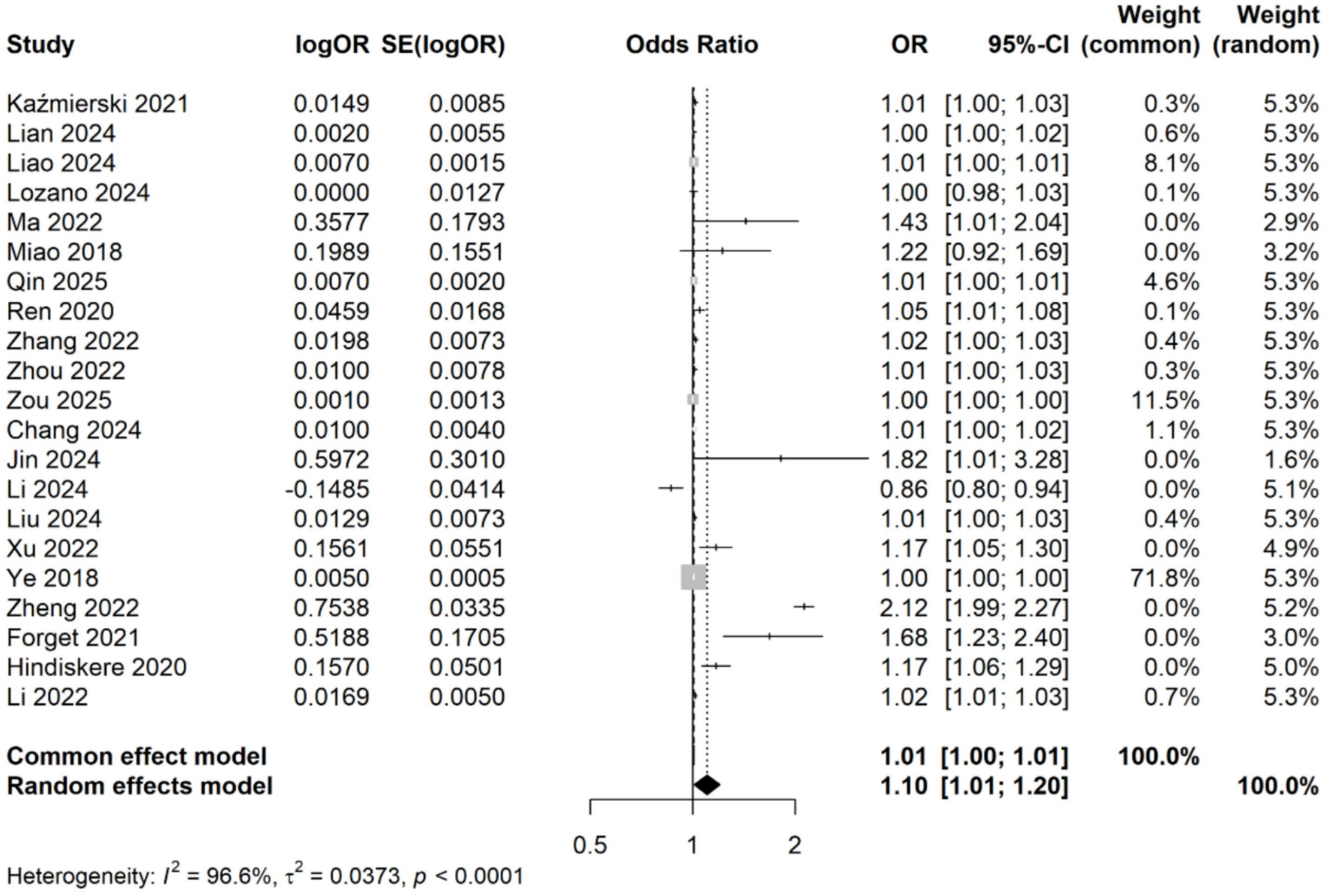
Forest plot of the meta-analysis of the association of CRP levels with delirium based on continuous data.

**Figure 3:**
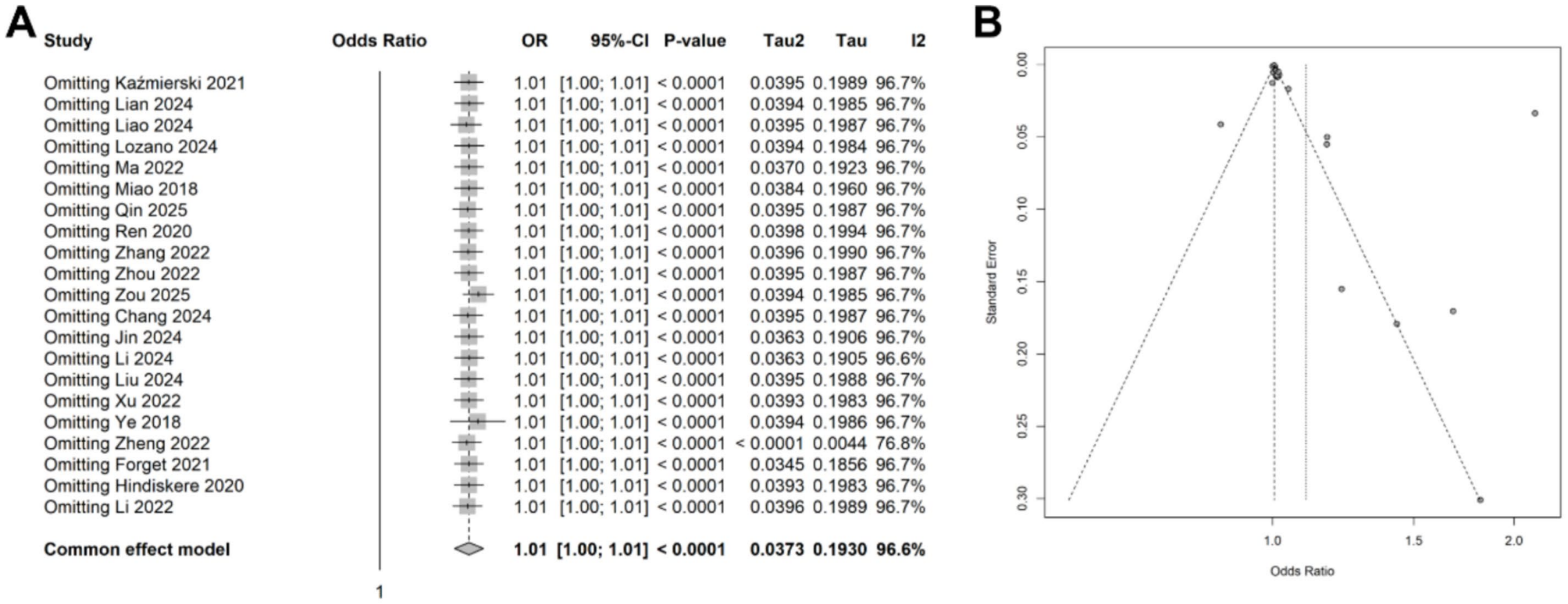
**(A)** Forest plot of the sensitivity analysis for the meta-analysis result of continuous data. **(B)** Funnel plots of the publication bias test in the meta-analysis.

A meta-analysis was conducted on 21 studies involving 5,006 patients to evaluate the association between CRP levels (as continuous variables) and delirium. Using a random-effects model due to significant heterogeneity (*I*^2^ = 96.6%, *p* < 0.001), we observed a statistically significant association between elevated CRP levels and increased delirium risk (OR: 1.10, 95% CI: 1.01–1.20, *p* = 0.030) (Figure 2). Sensitivity analyses demonstrated that the overall association remained stable after sequentially excluding each study, indicating that no single study unduly influenced the findings (Figure 3A). Funnel plots were largely symmetrical, and Egger’s regression test yielded *p* = 0.054 (Figure 3B), indicating borderline evidence of potential small-study effects or publication bias.

### Meta-regression and subgroup analysis for the association of CRP levels with delirium

**Figure 4:**
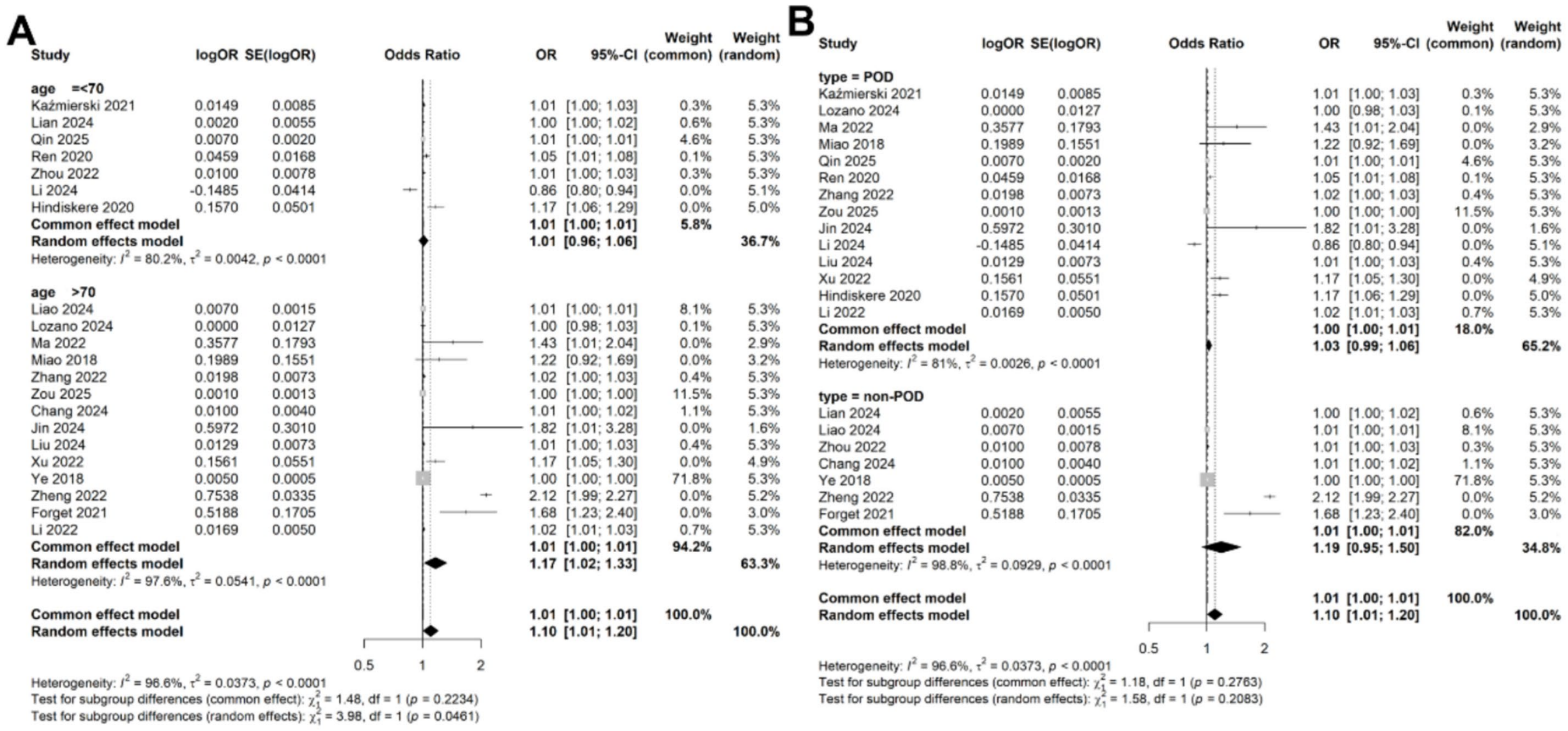
Subgroup analysis for the meta-analysis of the association of CRP levels with delirium based on continuous data in **(A)** different age and **(B)** disease types.

Given the significant heterogeneity observed across studies, we performed a meta-regression to identify potential sources of this variability. Four variables, study location (China vs. other countries; *p* = 0.354), study design (prospective vs. retrospective; *p* = 0.115), disease type (POD vs. non-POD; *p* = 0.281), patient age (>70 years vs. <70 years; *p* = 0.147), and diagnostic criteria (CAM vs. DSM; *p* = 0.154) were included in the meta-regression analysis. However, none of these variables significantly accounted for the heterogeneity (Table 2). Subsequently, subgroup analyses based on patient age and disease types confirmed that the association between CRP levels and delirium remained consistent across different subgroups (Figures 4A,B).

**Table 2:** Meta-regression for the variables affect the pooled effect in continuous data.

Variables	Num. of study	*R*^2^ value	*P*-value
Age (>70 years vs. <70 years)	14 vs. 7	2.23%	0.147
Location (China vs. other countries)	17 vs. 4	0	0.354
Study design (prospective vs. retrospective)	12 vs. 9	4.79%	0.115
Disease type (POD vs. non-POD)	13 vs. 7	1.29%	0.281
Diagnostic criteria (CAM vs. DSM)	9 vs. 12	1.36%	0.154

### Association of CRP levels with delirium based on categorical data

**Figure 5:**
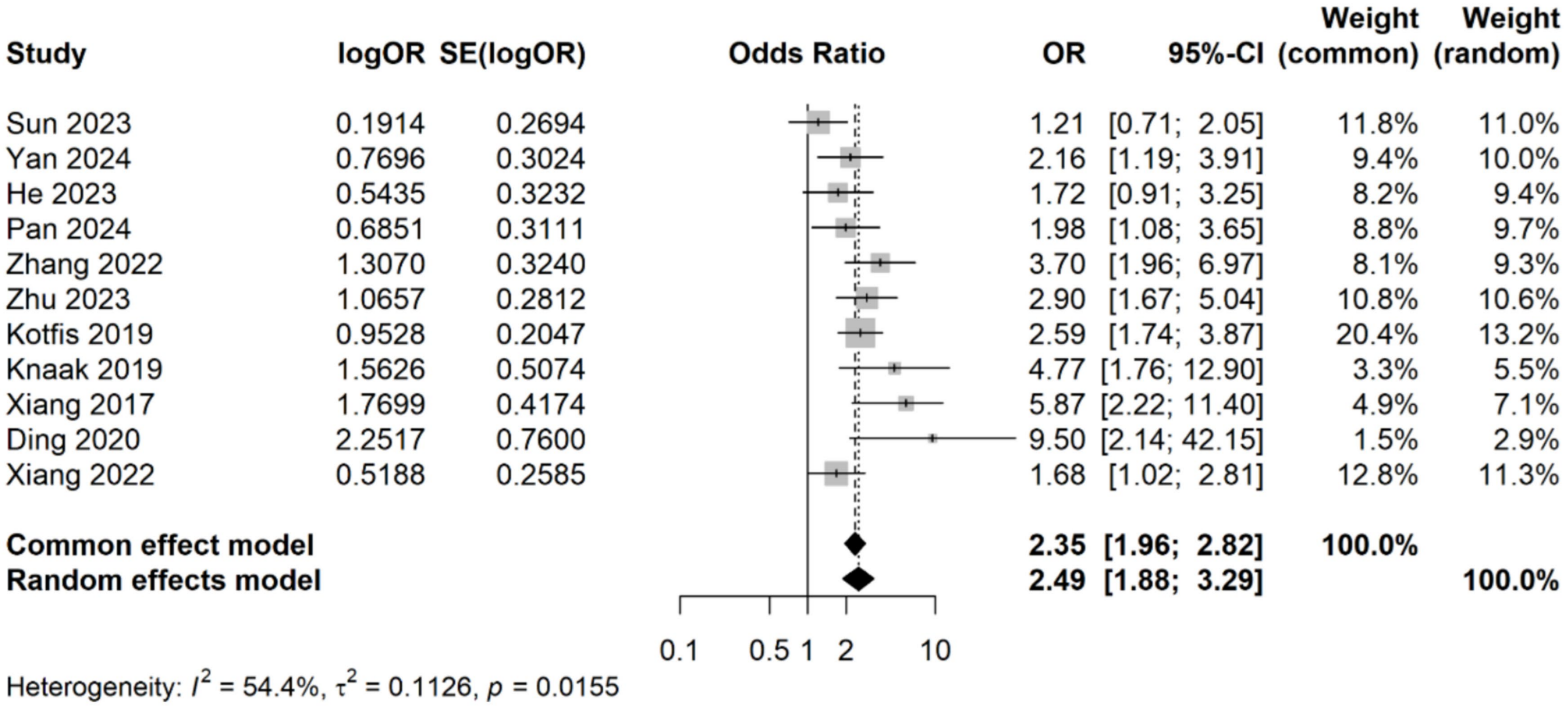
Forest plot of the meta-analysis of the association of CRP levels with delirium based on categorical data.

**Figure 6:**
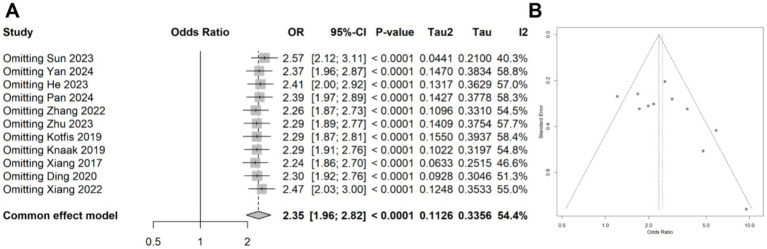
**(A)** Forest plot of the sensitivity analysis for the meta-analysis result of categorical data. **(B)** Funnel plots of the publication bias test in the meta-analysis.

A meta-analysis was conducted on 11 studies involving 3,996 patients, which categorized CRP levels into high and low groups based on predefined thresholds. The pooled data revealed a significant association between elevated CRP levels and increased risk of delirium (OR: 2.49, 95% CI: 1.88–3.29, *p* < 0.001), with significantly statistical heterogeneity among the studies (*I*^2^ = 54.4%, *p* = 0.015) (Figure 5). Sensitivity analyses demonstrated that the overall association remained stable after sequentially removing each study, confirming the consistent of this finding (Figure 6A). Funnel plots were largely symmetrical, and Egger’s test suggested no evidence of publication bias (*p* = 0.643) (Figure 6B).

### Meta-regression and subgroup analysis for the association of CRP levels with delirium

**Figure 7:**
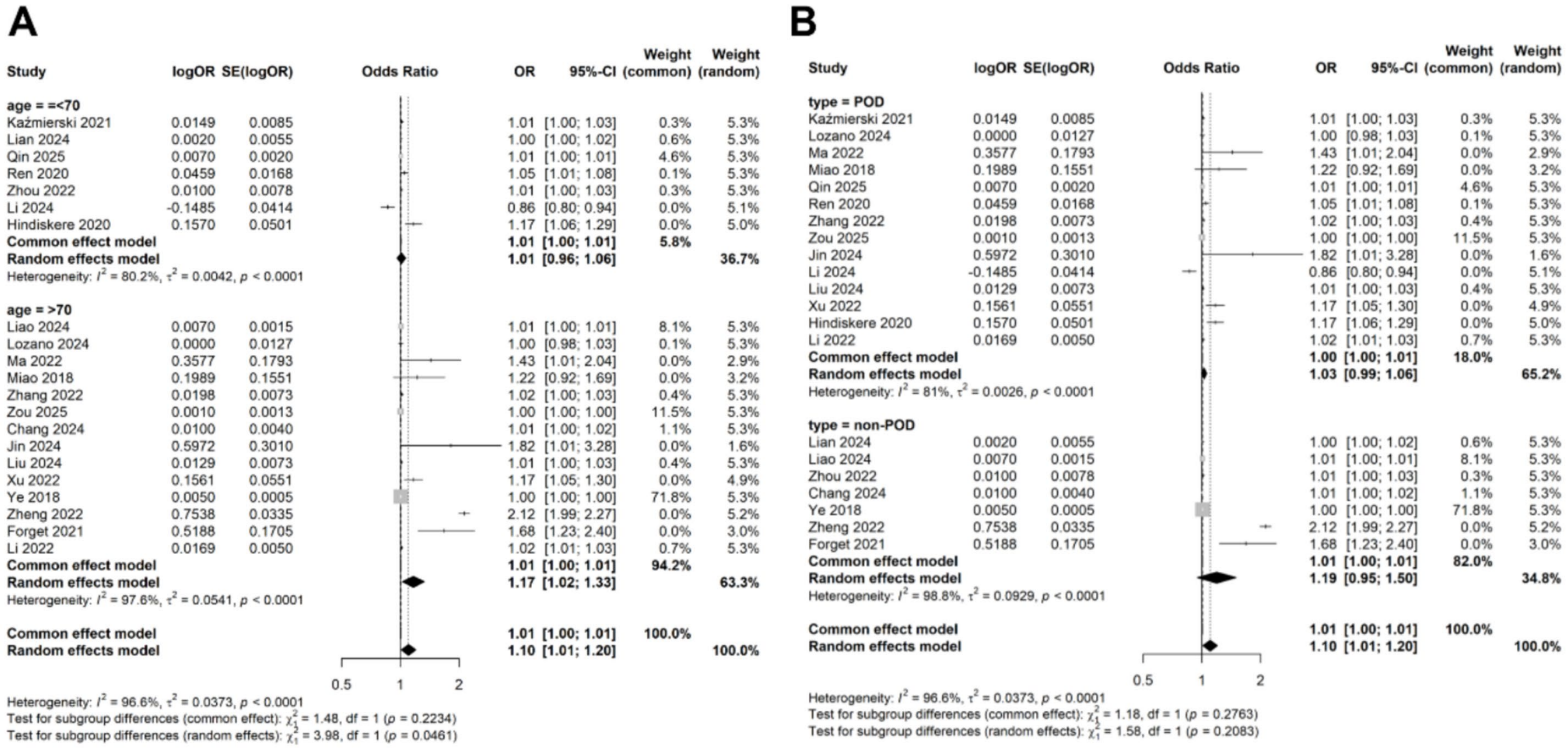
Subgroup analysis for the meta-analysis of the association of CRP levels with delirium based on categorical data in **(A)** different age and **(B)** disease types.

Due to the observed heterogeneity in the categorical data analysis, we again performed a meta-regression using the same four variables: study location, study design, disease type, patient age, and diagnostic criteria. The results indicated that none of these variables significantly contributed to the heterogeneity (age: *p* = 0.126; location: 0.723; study design: 0.163; disease type: 0.215; diagnostic criteria: 0.168; Table 3). Subgroup analyses based on patient age and disease type showed that the association between CRP levels and delirium remained consistent across different subgroups (Figures 7A,B).

**Table 3:** Meta-regression for the variables affect the pooled effect in categorical data.

Variables	Num. of study	*R*^2^ value	*P*-value
Age (>70 years vs. <70 years)	9 vs. 2	2.23%	0.126
Location (China vs. other countries)	9 vs. 2	0	0.723
Study design (prospective vs. retrospective)	3 vs. 2	5.75%	0.163
Disease type (POD vs. non-POD)	8 vs. 3	1.29%	0.215
Diagnostic criteria (CAM vs. DSM)	6 vs. 5	1.36%	0.168

## Discussion

The CRP is a biomarker of systemic inflammation, it has gained increasing attention for its potential role in predicting delirium (42). In this meta-analysis, we comprehensively examined the relationship between serum CRP levels and delirium by analyzing data from 32 studies involving 9,944 patients. Both continuous and categorical analyses revealed a significant association between elevated serum CRP levels and an increased risk of delirium. These findings suggest that CRP may play a critical role in the pathophysiology of delirium. However, significant heterogeneity was observed among the included studies. Meta-regression analysis failed to identify patient age, study location, study design, disease type, or diagnostic criteria as sources of heterogeneity. Subgroup analyses based on age and disease type further confirmed that the association between CRP levels and delirium remained consistent with the main results. Collectively, these findings underscore the potential of serum CRP as a biomarker for delirium and highlight the need for future research to explore underlying biological mechanisms and evaluate its utility for early prediction and intervention.

The inflammatory response reflected by elevated CRP levels may contribute to delirium through multiple mechanisms, including disruption of neurotransmitter function and blood–brain barrier integrity (43). Previous studies have demonstrated the predictive value of CRP in specific clinical contexts. For example, elevated preoperative CRP levels were significantly associated with an increased risk of POD. Patients with high CRP levels exhibited a 4.8-fold higher risk of developing POD compared to those with lower levels, underscoring the specificity of CRP as a predictor of delirium (18). Similarly, in stroke patients, serum CRP levels measured within 24 h of symptom onset were linked to an increased risk of delirium. Notably, CRP acted as an independent predictor of delirium even after adjusting for clinical factors such as age, atrial fibrillation, diabetes mellitus, and hemorrhagic stroke (44). Despite these findings, inconsistent results have been reported in some studies. For instance, Brattinga et al. (45) conducted a prospective study that failed to demonstrate an association between CRP levels and POD. Similarly, a recent study reported that serum CRP was not a significant predictor of POD after multivariate regression analysis (25). These discrepancies may arise from differences in study populations, CRP measurement timing, or confounding variables. Such inconsistencies highlight the need for validation of CRP’s predictive value in larger, well-designed prospective cohorts.

The substantial heterogeneity observed in our meta-analysis, while the meta-regression did not identify sources of heterogeneity, which might due to limited number of studies per subgroup. We speculated that the heterogeneity may stem from multiple methodological and clinical factors. First, variations in study design contributed significantly to inconsistency. Differences in the timing of CRP measurements, such as timing of CRP measurement (pre- vs. post-onset) (19, 25), thresholds for defining elevated CRP (2 mg/L vs. 48 mg/L) (31, 32), and differences in delirium assessment tools (CAM vs. DSM), critically influenced the interpretation of inflammatory burden. Second, population diversity introduced variability, particularly between surgical and non-surgical cohorts. For example, surgical patients often exhibit transient CRP spikes due to tissue trauma, whereas critically ill medical patients may experience sustained elevations due to sepsis or organ failure, complicating direct comparisons (9). Third, heterogeneity in delirium assessment tools likely affected the accuracy of case identification (46). Fourth, uncontrolled confounding factors, such as age, baseline cognitive impairment, and comorbidities, which independently elevate CRP, were inconsistently adjusted across studies, obscuring delirium-specific inflammatory signals (47). Finally, technical variability in CRP assays, including the use of high-sensitivity versus standard methods, may have exacerbated measurement discrepancies, particularly in studies detecting low-grade inflammation (48). These multifaceted sources of heterogeneity highlight the urgent need for standardized protocols in future research to clarify CRP’s role in delirium pathogenesis.

This meta-analysis offers several notable strengths. First, it synthesizes data from diverse clinical populations, encompassing a large sample size that enhances the generalizability of findings across various delirium etiologies. By employing rigorous subgroup analyses stratified by delirium subtypes (postoperative vs. infection-related), the study mitigates heterogeneity inherent in prior fragmented evidence. Additionally, we pooled data using both continuous and categorical approaches, with both analyses consistently confirming the association between CRP levels and delirium. This dual approach strengthens the consistent of our findings. However, the *p* value of Egger’s test for dichotomous outcomes was 0.054, suggested inconclusive evidence of publication bias, and might affect the reliable of the results. Furthermore, the association between elevated CRP and delirium risk is statistically significant, the effect size (OR = 1.10) may have limited utility as a standalone predictive biomarker in clinical practice. CRP should be interpreted alongside other risk factors rather than in isolation.

Despite these strengths, several limitations warrant consideration. First, all the included studies were observational design, due to the inability to infer causality from observational data, reverse causality (delirium causing CRP elevation) should be improved. Second, residual heterogeneity persists due to variability in CRP measurement timing (pre- vs. post-delirium onset) and inconsistent adjustment for inflammatory confounders, such as which may attenuate causal inferences. Third, the predominance of observational studies limits the ability to draw definitive conclusions about CRP mechanistic role in delirium pathogenesis, as reverse causality cannot be ruled out. Fourth, the predominance of studies from China (26/32, approximately 85% of patients), which may limit the generalizability of our findings to other ethnic or geographic populations. Fifth, several key confounders (age, baseline cognitive impairment, dementia, severity of illness, and medication use) is inconsistent across included studies, these confounders may also lead to the heterogeneity of the results. To address these gaps, future research should prioritize prospective longitudinal designs with standardized CRP measurement protocols. Such studies should also incorporate comprehensive adjustments for confounding variables and explore diverse populations to validate and extend these findings. Additionally, mechanistic studies are needed to elucidate the biological pathways linking CRP-mediated inflammation to delirium, potentially paving the way for targeted interventions.

## Conclusion

This meta-analysis demonstrates that elevated serum CRP levels are significantly but modestly associated with delirium risk in diverse clinical populations. Given the observational nature of the included studies and high heterogeneity, these findings support CRP as a correlate, rather than a causal mediator, of delirium-related inflammation. The small effect size for continuous CRP suggests limited utility as a standalone predictor, though it may contribute to multivariable risk stratification models. Future prospective studies with standardized protocols are needed to clarify its clinical applicability.

## Data Availability

The original contributions presented in the study are included in the article/Supplementary material, further inquiries can be directed to the corresponding author.
