# Supplementary material for: Meta-analysis for the associations of serum C-reactive protein with delirium risk
Source: Front Neurol. 2026 Jan 29;17:1728476. doi: 10.3389/fneur.2026.1728476 (PMC12893975; doi:10.3389/fneur.2026.1728476)
Supplement: Supplementary file 1 [file Table_1.DOCX]

**Supplement files**

**Table S1. Full Search Strategies for Each Database**

| **Database** | **Search Strategy (as of March 1, 2025)** |
| --- | --- |
| **PubMed** | ("Delirium"[MeSH Terms] OR "Acute Confusional State"[Title/Abstract] OR deliri*[Title/Abstract])AND("C-Reactive Protein"[MeSH Terms] OR CRP[Title/Abstract] OR "C reactive protein"[Title/Abstract])AND("Humans"[MeSH Terms]) |
| **EMBASE (via Ovid)** | (delirium/ OR 'acute confusional state'.mp. OR deliri*.mp.)AND(exp C reactive protein/ OR CRP.mp. OR 'C reactive protein'.mp.)AND(limit to (human)) |
| **Ovid MEDLINE** | (Delirium/ OR "Acute Confusional State".ti,ab. OR deliri*.ti,ab.)AND(exp C-Reactive Protein/ OR CRP.ti,ab. OR "C reactive protein".ti,ab.)AND(exp Humans/) |
| **CNKI (China National Knowledge Infrastructure)** | 主题：("谵妄" OR "急性脑病综合征")AND主题：("C反应蛋白" OR "CRP")（检索字段：主题；文献类型：学术期刊；语言：中文） |
| **CBM (Chinese Biomedical Literature Database)** | (“谵妄”[常用字段:智能] OR “急性意识模糊”[常用字段:智能])AND(“C反应蛋白”[常用字段:智能] OR “CRP”[常用字段:智能])限定条件：人类 |

Table S2. Newcastle-Ottawa Scale (NOS) Quality Assessment of the Included Studies

| study | Representativeness of the exposed cohort | Selection of the nonexposed cohort | Ascertainment of exposure | Demonstration that outcome of interest was not present at the start of study | Study controls for age, sex, marital status | Study controls for any additional factor | Assessment of outcome | Was follow up long enough for outcomes to occur | Adequacy of follow-up of cohorts | overall score |
| --- | --- | --- | --- | --- | --- | --- | --- | --- | --- | --- |
| Author | 1 | 1 | 1 | 1 | 1 | 1 | 1 | 1 | 1 | 9 |
| Kaźmierski | 1 | 1 | 1 | 1 | 1 | 1 | 1 | 1 | 1 | 9 |
| Klimiec | 1 | 1 | 1 | 1 | 1 | 1 | 1 | 1 | 1 | 9 |
| Lian | 1 | 1 | 1 | 1 | 1 | 1 | 1 | 1 | 1 | 9 |
| Liao | 1 | 1 | 1 | 1 | 1 | 1 | 1 | 1 | 1 | 9 |
| Lozano | 1 | 1 | 1 | 1 | 1 | 1 | 1 | 1 | 1 | 9 |
| Ma | 1 | 1 | 1 | 1 | 1 | 1 | 1 | 1 | 1 | 9 |
| Miao | 1 | 1 | 1 | 1 | 1 | 1 | 1 | 1 | 1 | 9 |
| Qin | 1 | 1 | 1 | 1 | 1 | 1 | 1 | 1 | 1 | 9 |
| Ren | 1 | 1 | 1 | 1 | 1 | 1 | 1 | 1 | 1 | 9 |
| Sun | 1 | 1 | 1 | 1 | 1 | 1 | 1 | 1 | 0 | 8 |
| Yan | 1 | 1 | 1 | 1 | 0 | 1 | 1 | 1 | 1 | 8 |
| Zhang | 1 | 1 | 1 | 1 | 1 | 1 | 1 | 1 | 1 | 9 |
| Zhou | 1 | 1 | 1 | 1 | 1 | 1 | 1 | 1 | 1 | 9 |
| Zou | 1 | 1 | 1 | 1 | 1 | 1 | 1 | 1 | 1 | 9 |
| Chang | 1 | 1 | 1 | 1 | 1 | 1 | 1 | 1 | 1 | 9 |
| He | 1 | 1 | 1 | 1 | 1 | 1 | 1 | 1 | 1 | 9 |
| Jin | 1 | 1 | 1 | 1 | 1 | 1 | 1 | 1 | 1 | 9 |
| Li | 1 | 1 | 1 | 1 | 1 | 1 | 1 | 1 | 1 | 9 |
| Liu | 1 | 1 | 1 | 1 | 1 | 1 | 1 | 1 | 1 | 9 |
| Pan | 1 | 1 | 0 | 1 | 1 | 1 | 1 | 1 | 0 | 7 |
| Xu | 1 | 1 | 1 | 1 | 1 | 1 | 1 | 1 | 1 | 9 |
| Ye | 1 | 1 | 1 | 1 | 1 | 1 | 1 | 1 | 1 | 9 |
| Zhang | 1 | 1 | 1 | 1 | 1 | 1 | 1 | 1 | 0 | 8 |
| Zheng | 1 | 1 | 1 | 1 | 1 | 1 | 1 | 1 | 1 | 9 |
| Zhu | 1 | 1 | 1 | 1 | 1 | 1 | 1 | 1 | 1 | 9 |
| Forget | 1 | 1 | 1 | 1 | 1 | 1 | 1 | 1 | 1 | 9 |
| Hindiskere | 1 | 1 | 1 | 1 | 1 | 1 | 1 | 1 | 1 | 9 |
| Kotfis | 1 | 1 | 1 | 1 | 1 | 1 | 1 | 1 | 1 | 9 |
| Knaak | 1 | 1 | 1 | 1 | 1 | 1 | 1 | 1 | 1 | 9 |
| Xiang | 1 | 1 | 1 | 1 | 1 | 1 | 1 | 1 | 0 | 8 |
| Ding | 1 | 1 | 1 | 1 | 1 | 1 | 1 | 1 | 1 | 9 |
| Xiang | 1 | 1 | 1 | 1 | 1 | 1 | 1 | 1 | 1 | 9 |
| Ding | 1 | 1 | 1 | 1 | 1 | 1 | 1 | 1 | 1 | 9 |
| Li | 1 | 1 | 1 | 1 | 1 | 1 | 1 | 1 | 1 | 9 |
